# Supplementary material for: Megafire affects stream sediment flux and dissolved organic matter reactivity, but land use dominates nutrient dynamics in semiarid watersheds
Source: PLoS One. 2021 Sep 23;16(9):e0257733. doi: 10.1371/journal.pone.0257733 (PMC8460006; doi:10.1371/journal.pone.0257733)
Supplement: S1 Fig — We created the maps with ArcGIS Pro (ESRI) using open source basemap layers from the global GIS user community (the USGS National Map and Earth Resources Observation and Science Center). (DOCX) [file pone.0257733.s001.docx]

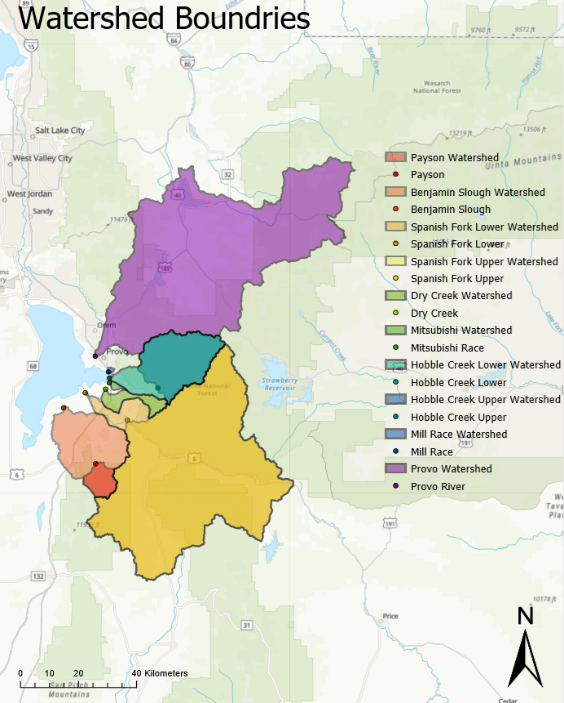

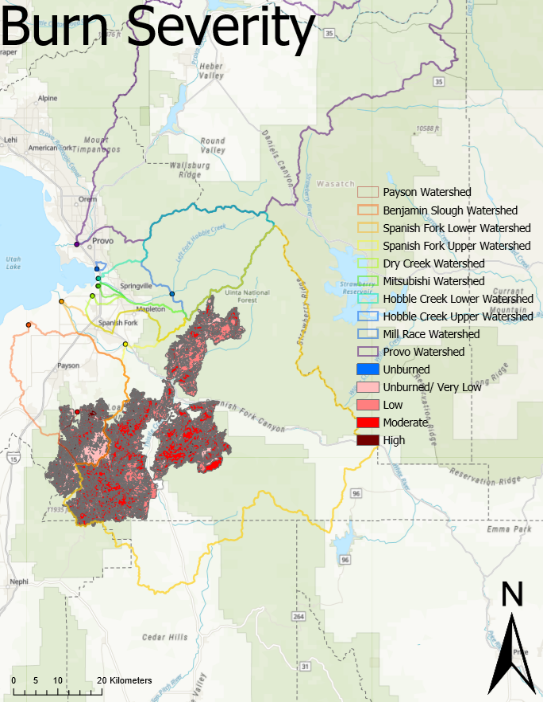


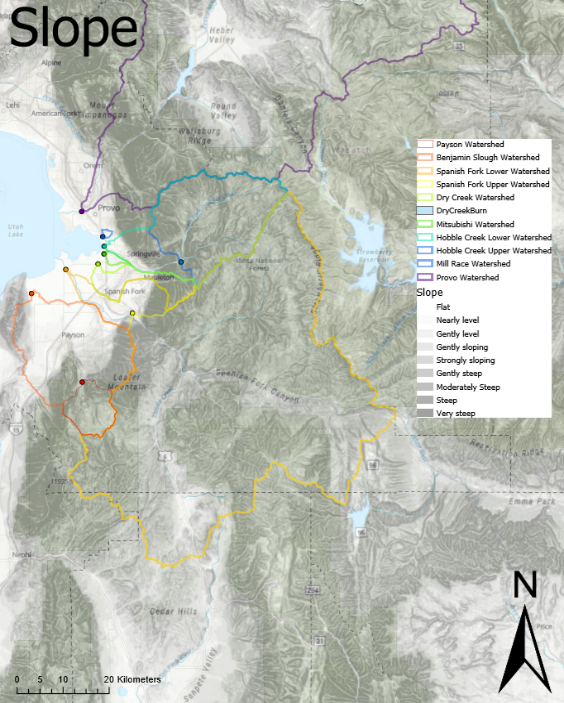

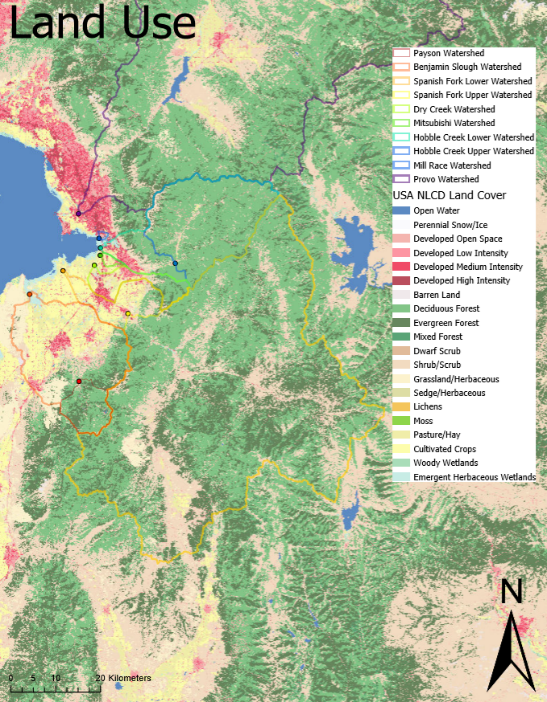


**Figure S1.** Detailed maps of the study watersheds, burn scar severity, topography, and land use. We created the maps with ArcGIS Pro (ESRI) using open source basemap layers from the global GIS user community (the USGS National Map and Earth Resources Observation and Science Center).
