# Supplementary material for: Megafire affects stream sediment flux and dissolved organic matter reactivity, but land use dominates nutrient dynamics in semiarid watersheds
Source: PLoS One. 2021 Sep 23;16(9):e0257733. doi: 10.1371/journal.pone.0257733 (PMC8460006; doi:10.1371/journal.pone.0257733)
Supplement: S2 Fig — There was insufficient sample size to The boxplots represent the median and its 95% confidence interval (the notches), the interquartile range (IQR), and points beyond 1.5 times the IQR. (DOCX) [file pone.0257733.s002.docx]

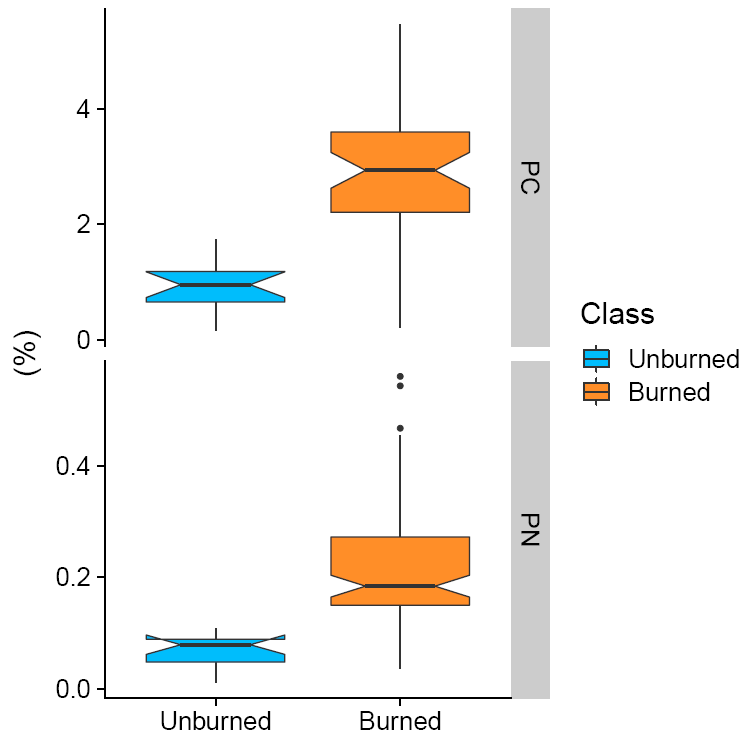


**Figure S2.** Particulate organic carbon and particulate nitrogen content (%) based on elemental analysis of material that did not pass through a glass fiber filter with an effective pore size of 0.7 µm. There was insufficient sample size to The boxplots represent the median and its 95% confidence interval (the notches), the interquartile range (IQR), and points beyond 1.5 times the IQR.
