# Supplementary material for: Megafire affects stream sediment flux and dissolved organic matter reactivity, but land use dominates nutrient dynamics in semiarid watersheds
Source: PLoS One. 2021 Sep 23;16(9):e0257733. doi: 10.1371/journal.pone.0257733 (PMC8460006; doi:10.1371/journal.pone.0257733)
Supplement: S3 Fig — Linear fit lines and 95% confidence intervals shown when p < 0.05. (DOCX) [file pone.0257733.s003.docx]

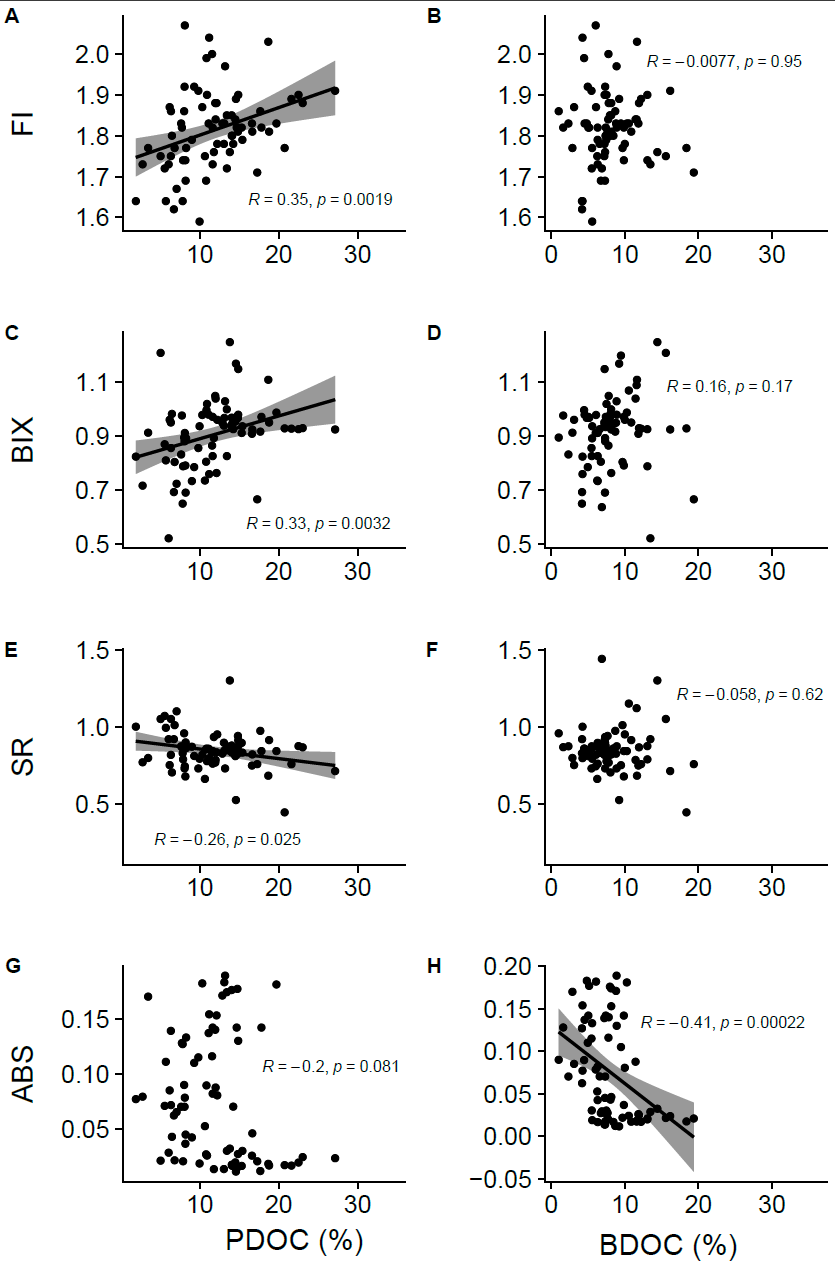


**Figure S3**. Individual pairwise comparisons of optical properties and biodegradable and photodegradable dissolved organic carbon (BDOC and PDOC). Linear fit lines and 95% confidence intervals shown when p < 0.05.
