# Supplementary material for: Megafire affects stream sediment flux and dissolved organic matter reactivity, but land use dominates nutrient dynamics in semiarid watersheds
Source: PLoS One. 2021 Sep 23;16(9):e0257733. doi: 10.1371/journal.pone.0257733 (PMC8460006; doi:10.1371/journal.pone.0257733)
Supplement: S4 Fig — (DOCX) [file pone.0257733.s004.docx]

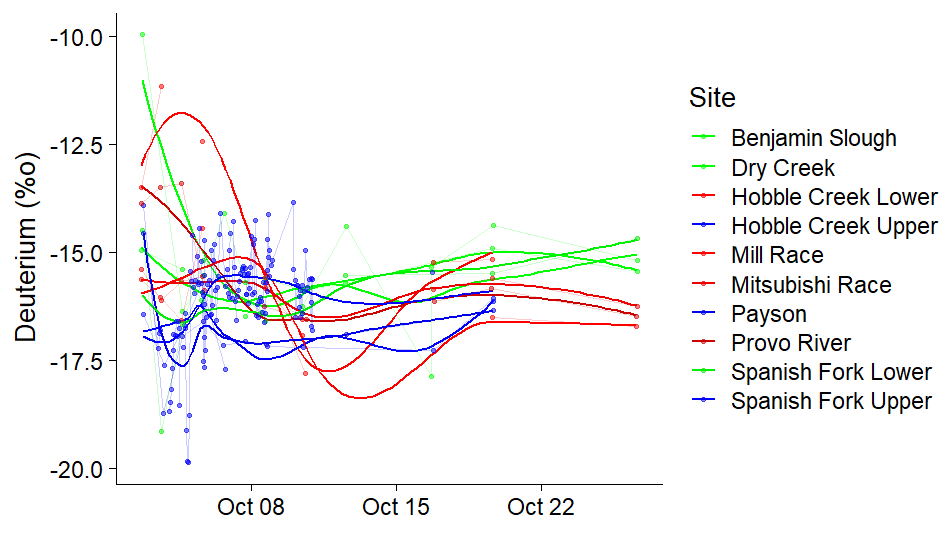


**Figure S4.** Time series of deuterium values for all the sites individually and the smoothed means for agricultural (green), urban (red), and natural (blue) sites.
